# Highly porous regenerated cellulose hydrogel and aerogel prepared from hydrothermal synthesized cellulose carbamate

**DOI:** 10.1371/journal.pone.0173743

**Published:** 2017-03-15

**Authors:** Sinyee Gan, Sarani Zakaria, Chin Hua Chia, Ruey Shan Chen, Amanda V. Ellis, Hatika Kaco

**Affiliations:** 1 School of Applied Physics, Faculty of Science and Technology, Universiti Kebangsaan Malaysia, Bangi, Selangor, Malaysia; 2 Department of Chemical Engineering, Melbourne University, Parkville, Melbourne, VIC, Australia; Institute of Materials Science, GERMANY

## Abstract

Here, a stable derivative of cellulose, called cellulose carbamate (CC), was produced from Kenaf (Hibiscus cannabinus) core pulp (KCP) and urea with the aid of a hydrothermal method. Further investigation was carried out for the amount of nitrogen yielded in CC as different urea concentrations were applied to react with cellulose. The effect of nitrogen concentration of CC on its solubility in a urea-alkaline system was also studied. Regenerated cellulose products (hydrogels and aerogels) were fabricated through the rapid dissolution of CC in a urea-alkaline system. The morphology of the regenerated cellulose products was viewed under Field emission scanning electron microscope (FESEM). The transformation of allomorphs in regenerated cellulose products was examined by X-ray diffraction (XRD). The transparency of regenerated cellulose products was determined by Ultraviolet–visible (UV–Vis) spectrophotometer. The degree of swelling (DS) of regenerated cellulose products was also evaluated. This investigation provides a simple and efficient procedure of CC determination which is useful in producing regenerated CC products.

## Introduction

Cellulose is the most abundant renewable biopolymer with excellent properties available in worldwide and has a broad range of properties such as biocompatibility, recyclable, outstanding mechanical strength, adjustable optical appearance, great thermostabilization, hydrophilicity, good sorption capacity, nontoxic etc. [1–3]. This natural linear polysaccharide has been primarily employed in wide applications such as textile, drug delivery, water treatment, construction, pharmaceutical, packaging, agriculture, pulp and paper industry [4]. Cellulose is a linear, polydisperse and syndiotactic polymer, as the anhydroglucopyranose monomeric repeat units (AGU) are covalently linked via acetal functions between the equatorial OH group of C4 and the C1 carbon [5]. Natural cellulose is difficult to process due to its insolubility in alcohol, water, and other common solvents. Therefore in order to use cellulose in common industrial processes it must be first derivitized to facilitate solubility in an appropriate solvent.

A great deal of attention has been given to cellulose carbamate (CC), a derivative of cellulose. CC is easily dissolvable in aqueous alkaline solutions and organic solvents [6]. As a result CC can be processed in order to place viscose method for the cellulose fiber production via conventional wet-spinning techniques. It is also more resistant to microbial attacks and enzymatic cleavage compare to native cellulose [6]. Furthermore, regenerated CC fibers are more biodegradable than viscose fibers [7] and as such can be used in a broad range of applications related to medical and healthcare hygiene as well as heavy metal adsorption [4]. This is primarily due to its microbiostatic properties and high water absorbency.

Various methods have been used to synthesize CC, however, the more conventional methods require severe conditions such as long reaction times, organic solvents, catalysts, high temperatures and pre-ripened cellulose starting materials [8,9]. Nada et al. [8] have mercerized cotton linter using sodium hydroxide under reflux system before reacting with urea. Other work has focused on an alternative route where cellulose is activated by sodium hydroxide and ammonia solution using the kneader technique [10,11]. The main role of ammonia in this system is to bring the structural changes in cellulose by swelling the fiber and facilitate its reaction with urea [12]. The limitation of this process is the use of large amounts of reagents. Thus, more recently cellulose has been reacted with only urea with reaction time less than an hour with the aid of microwave radiation both with [13,14] and without polar media [15]. Vo et al. [4] have synthesized CC in a non-alkali solution using polyethylene glycol, lithium chloride and urea at temperatures up to 220°C for 2 min. While Yin and Shen [16] have prepared CC with the aid of ethanol and supercritical carbon dioxide at 50°C for 6 h under 18 MPa pressure. Therefore, the hydrothermal method was employed in the present paper in order to produce CC effectively using urea and water only without any catalyst, organic solvent, reagent or activation using ammonia.

Various gels can be formed from the dissolution of cellulose, including, but not limited to, hydrogels and aerogels. Cellulose hydrogels are typically achieved through the dissolution of cellulose in various type of solvents which induces the physical cross-linking of the cellulose hydroxyl groups, forming hydrogen bonded networks [17]. Cellulose hydrogels have been widely employed in different applications such as drug delivery systems, cool dewatering, hygienic products, sealing, pharmaceuticals, separation of biomolecules or cells, food additives, tissue engineering and biomedical applications [18].

Aerogels are highly porous solids that contain gas (commonly air) within their pores or networks. They are extremely light with a large inner surface area and have low heat conductivity. Hence, they are very useful in many applications such as particle filters, particle trappers, catalyst supports and heat insulators [19]. Cellulose aerogels are typically made through a solvent exchange and freeze-drying process of hydrogels and importantly this process avoids shrinkage of the hydrogel and yields aerogel porosities of up to 99.5% [20].

There were limited literatures reported on preparation and characterization of regenerated cellulose hydrogel and aerogel from the synthesized CC. Therefore, in this work, CC was made using various urea concentrations and its regenerated cellulose products were produced. Particular emphasis was placed on evaluating the influence of nitrogen concentration of CC formed via hydrothermal method on its solubility in urea-alkaline solution as well as the properties of its regenerated hydrogels and aerogels.

## Experimental

### Materials

Kenaf core was supplied by the Forest Research Institute Malaysia (FRIM). Analytical grade lithium hydroxide monohydrate (LiOH∙H_2_O) (98%), epichlorohydrin, sulphuric acid (98.8%) and urea (99%) were purchased from Sigma-Aldrich, Malaysia and used as received.

### Methods

#### Fabrication of kenaf core pulp

The kenaf core was soda pulped in Forest Research Institute Malaysia (FRIM) in a digester with 25% NaOH concentration at 170°C for 150 min. The kenaf core was soda pulped and the resulting kenaf core pulp (KCP) was bleached using a four stage bleaching method (DEED), as described in our previous work [21,22]. The bleached KCP was then dried at 105°C for 24 h.

#### Synthesis of kenaf CC

KCP (4 g) was milled and immersed into an aqueous urea solution at urea/KCP ratio of 2:1, 4:1 and 6:1. Each KCP mixture was stirred at ambient temperature for 30 min to produce a homogeneous mixture. The KCP mixture was then placed into an autoclave and immersed into an oil bath at 140°C for 3 h. For each mixture of different urea/KCP ratios, after the reaction time was reached, the autoclave was removed from the hot oil bath and immersed instantly in a cold water bath to stop the reaction. The obtained CC was washed with distilled water several times to remove the excessive urea and production yield was in the range of 84 to 91 wt%. The CC was then dried in a vacuum oven and stored at room temperature. The CC sample prepared using an urea/KCP ratio of 2, 4 and 6 was denoted as CC-2, CC-4 and CC-6, respectively.

#### Preparation of CC hydrogels and aerogels

KCP, CC-2, CC-4 or CC-6 (4 wt%) was dissolved in aqueous LiOH/urea solution (weight ratio = 4.6:15) at -13°C low temperature. According to the rapid dissolution method, resulting mixture was stirred rapidly for 5 min to obtain the homogeneous cellulose solution compared to pre-cooled method that required several cooling process. The viscosity of the cellulose solution was around 42000 cP. The solution was then centrifuged at 10000 rpm for 5 min at 5°C to remove any air bubbles and to separate the cellulose solution and undissolved cellulose. The undissolved cellulose were washed and dried in a vacuum oven at 80°C for 12 h in order to determine the percentage of cellulose solubility.

In a typical hydrogel synthesis procedure, the cross-linking agent epichlorohydrin solution (5%) was added drop wise into the cellulose solution. The resulting viscous cellulose solution was stirred at room temperature for approximately 1 h until the hydrogel was formed. The hydrogel sample was then cleaned in a distilled water bath at room temperature for 3 days. The hydrogel formed using KCP was denoted kenaf core pulp hydrogel (KCPH) and the CC hydrogel were denoted CCH-2, CCH-4 and CCH-6. Finally, in order to produce cellulose aerogels, the hydrogel samples were freeze-dried for 48 h after the cleaning process. The cellulose aerogel was stored in a dry place for further characterization. The cellulose aerogels prepared by KCP were denoted kenaf core pulp aerogel (KCPA) and the CC aerogel samples were denoted CCA-2, CCA-4 and CCA-6.

#### Determination of nitrogen element percentage

The percentage of nitrogen in each CC sample was determined using an elemental analyzer, Interscience Flash EA 1112 series (Thermo Finnigan) with a TCD detector, CHNS—Porapack PQS column at 50–190°C.

#### Determination of degree of substitution

The nitrogen percentage was used to calculate the degree of substitution (DS) on cellulose by carbamate groups following equation [4]:

$$\text{DS}=\frac{162\times N\;(\%)}{14\;\times 100\;\text{-}\;43\times N\;(\%)}$$

where N (%) is the nitrogen content of modified cellulose determined by elemental analysis, 162 is the molecular weight of the anhydroglucose unit (AGU), 14 is the molecular mass of nitrogen atom and 43 is the net increment in the AGU for every substituted carbamate group.

#### Determination of solubility and concentration of cellulose solution

The solubility of 4 wt% of each cellulose sample (KCP, CC-2, CC-4 and CC-6) in LiOH/urea aqueous solution was calculated using Eq. below:

$$S=\frac{W_{o}-W}{W_{o}}\;x\;100\;\%$$

where *S* is the degree of dissolution for cellulose, *W* is the weight of undissolve cellulose residue and *W*_*o*_ is the original weight of the cellulose as discussed in our previous report Gan et al. [23].

Concentration of cellulose solution (Conc. CS) in weight percentage (wt %) in the LiOH/urea aqueous solvent was computed using following Eq:

$$\text{Conc}.\;\text{CS}=\text{S x}\;W_{o}=W_{o}\;\text{-}\;W$$

#### Fourier transform infrared (FT-IR) spectroscopy

In order to observe the functional groups in the samples FT-IR was performed on the raw KCP, CC and CCA samples using an FT-IR apparatus (Perkin Elmer Spectrum 400). The samples were analyzed in the range of 3000 cm^-1^ to 1400 cm^-1^ with 32 number of scans and resolution of measurement was set to 4 cm^-1^.

#### Determination of cellulose phase in aerogels

X-ray diffraction (XRD) was carried out to determine the phase of cellulose I or II in the KCP and aerogel samples. The XRD analysis was performed using a Bruker Axs D8 Advance diffractometer with CuKα radiation (λ = 0.15458 nm). The samples were scanned from a diffraction angle (2θ) range of 5 to 80°.

#### Swelling properties of the hydrogels

The CC hydrogels (CCH-2, CCH-4 and CCH-6) were immersed in distilled water bath at room temperature for 25 d until the absorption equilibrium was achieved. The weight of each sample was measured and the degree of swelling (DS) was calculated according to the following Eq.:

$$\text{DS}\;(\%)=\frac{W_{\text{s}}-W_{\text{d}}}{W_{\text{d}}}\times 100$$

Where *W*_d_ and *W*_s_ are the weights of the hydrogel before and after immersion in water for 25 d, respectively.

#### Transparency of the hydrogels

The cellulose hydrogels were cut into a thickness of 0.5 cm and the light transmittance through the hydrogels was measured using an Ultraviolet–Visible (UV–Vis) spectrophotometer at a wavelength ranging from 200 to 800 nm.

#### Morphology and porosity of aerogels

The morphology and porosity of the cellulose aerogels were observed using field emission scanning electron microscope (FESEM) (Zeiss/Supra 55VP) with an accelerating voltage at 10 kV. The freeze-dried CC aerogels were coated with gold before being subjected to FESEM examination. The samples were viewed under magnification of 50 X. The pore size of the cellulose aerogel was measured using SmartTiff software from Carl Zeiss Microscopy Limited in Cambridge, United Kingdom.

## Results and discussion

### Characterization of CC

**Fig 1:**
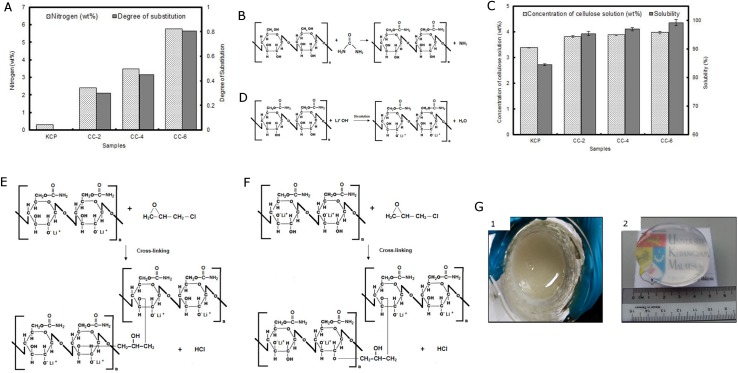
Characterization of CC. (A) Nitrogen concentration and degree of substitution of CC sample. (B) Chemical reaction scheme for carbamation process. (C) Solubility and concentration of cellulose solution. (D) Proposed chemical reaction of cellulose dissolved in LiOH/urea solvent. (E) Postulated chemical reaction for cellulose hydrogel cross-linking process (i). (F) Postulated chemical reaction for cellulose hydrogel cross-linking process (ii).(G) Photo of (1) cellulose solution (2) cellulose hydrogel.

As reported in our previous study, the molecular weight and degree of polymerization of KCP was 2.49 x 10^5^ and 3479, respectively [24]. The nitrogen (wt%) and degree of substitution of the CC samples (CC-2, CC-4, CC-6) are shown in Fig 1A. For the carbamation process, as the amount of urea added to the KCP was increased the nitrogen concentration and degree of substitution of the CC products (CC-2 to CC-6) increased. As the urea concentration increased, the substitution of carbonyl (C = O) and amine groups (-NH2) from the decomposition of urea into the cellulose was more sufficient. In other words, higher substitution of carbonyl and amine groups in cellulose can be obtained from higher concentration of urea supplied as more urea has been decomposed into carbonyl and amine groups. This indicates higher nitrogen concentration can be detected from the higher degree of substitution of amine groups in cellulose chain.

Fig 1B portrays the chemical reaction of cellulose and urea in the hydrothermal carbamation process for the formation of CC. During the carbamation process, urea is decomposed to isocyanic acids (consists of carbonyl (C = O) and amine groups (-NH_2_)) and ammonia gas (Fig 1B) as described in Nada et al. [8]. The isocyanic acids will then react with hydroxyl group in C6 position. The cellulose molecule is practically monofunctional in terms of aliphatic hydroxyl groups (OH-) [25]. The single glucose monomers in the cellulose are usually denoted as anhydroglucose units. There are three hydroxyl groups exist in the anhydroglucose unit of cellulose which is a primary in C6 and two secondary in C2 and C3 position. The C1 in cellulose possesses reducing properties whereas the C4 of cellulose is non-reducing end. Therefore, derivatization reactions target primarily substitutions of OH- groups in C6, C2 and C3 of cellulose. The relative substituent distribution among C2, C3 and C6 position are relying on the reaction condition [26].

Moreover, the rotational conformation of hydroxyl group on C6 can be modified. This shows a significant impact on the hydrogen bonding structure and crystallinity of cellulose [3]. The isocyanic acid will possibly react on C6 of AGU as shown in Fig 1B because C6 is a primary hydroxyl group which is highly accessible compares to C2 and C3 which is secondary hydroxyl group. In homogeneous conditions of cellulose solvent, the OH group in C6 is most reactive as it possesses lower steric hindrance as compares to secondary OH groups in C2 and C3 positions [26]. This has been confirmed by nuclear magnetic resonance testing in Klemm et al. [5] report, as the resonances of C6 (62–65 ppm) was decreased with the introduction of urea in the cellulose treatment. As C6 is primary hydroxyl, it has the most reactive reaction for esterification reaction [4]. This indicates that partial esterification preferably occurs at C6 position because the isocyanic acid tends to attack on OH group at C6 during carbamation process as agreed by Kotek [27].

Fig 1C describes the solubility of 4 wt% of each cellulose sample (KCP, CC-2, CC-4 and CC-6) in aqueous LiOH/urea solution via a rapid dissolution method. All CC samples with higher nitrogen concentration (Fig 1B) have higher average solubility than KCP (Fig 1C). As the nitrogen concentration of the CC samples increased, the solubility in aqueous LiOH/urea solution also increased. It was found that the CC-6 (9.7% nitrogen content) had the greatest solubility at 99.2%. This solubility is 12.7% greater than KCP (86.5%). The reason for this increased solubility is due to the disruption of the inter-molecular hydrogen bonding between the cellulose molecules after carbamate formation. This disruption allows the solvent to penetrate easily into the cellulose chains with subsequent expansion and swelling into the dissolution state.

Apart from this, the glycolic group (C–C cleavage between C2 and C3 with formation of a dialdehyde) plays an important role in the substitution reaction [25]. The reactivity among C6, C2 and C3 are different which offering selective reaction of cellulose for substitution of various functional groups [5]. Fig 1D presents the proposed chemical reaction of cellulose dissolved in LiOH/urea solvent. The C2 and C3 position are the favorable interaction sides for NaOH alkaline solvent as discussed in Wang [28], so the Li^+^ in this study will possibly react with C2 or C3 in anhydroglucose units during the cellulose dissolution process. Nevertheless, in comparison with C3, there is a highly acidic secondary hydroxyl group at C2 carbon in cellulose. The hydroxyl group in C2 possesses higher acidity and exhibits a more reactive site for etherification that allows the hydrogen atom on the hydroxyl group is easier to be replaced by other substituents than C3. Hence, the Li^+^ cation will most probably reacts with the C2 as reported by Klemm et al. [5] as illustrated in Fig 1D.

The first postulated chemical reaction for cellulose hydrogel in the cross-linking process between cellulose and ECH is shown in Fig 1E. Given the C2 (secondary hydroxyl) reacts with Li^+^ ions, the ECH will most likely attack the C3 (secondary hydroxyl) of cellulose. However, there is also the a minor possibility that the ECH may react with the C2 as the Li^+^ O^-^ ionic bond is not very stable in the cellulose chain. In addition, the chemical reaction of isocyanic acid, Li^+^ ions or ECH may not fully take place in the cellulose chain as the inter- and intra- hydrogen bonding in the cellulose will overshadow the cellulose chain and hinder the chemical reaction [5]. Therefore, Fig 1F illustrates the second postulated chemical reaction for cellulose hydrogel in the cross-linking process between cellulose and ECH. The Fig 1G gives a picture of cellulose solution before gelation and regenerated cellulose hydrogel.

### Swelling properties of cellulose hydrogels

**Fig 2:**
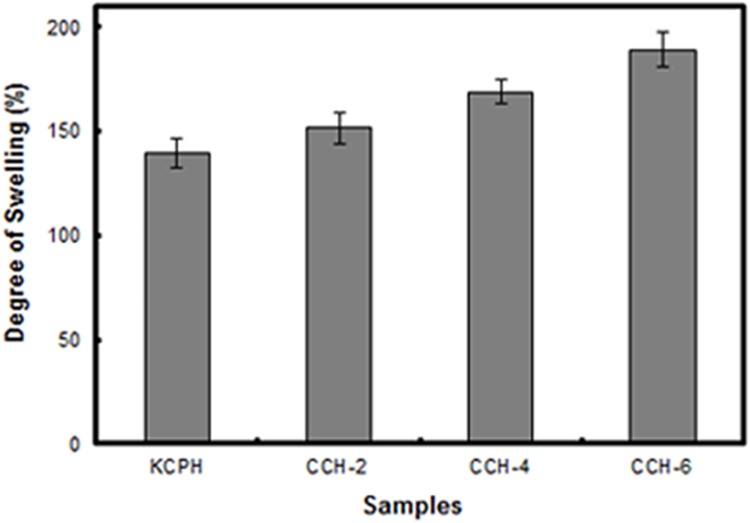
Degree of swelling of regenerated hydrogel.

The degree of swelling (%) of the regenerated cellulose hydrogels (KCPH, CCH-2, CCH-4 and CCH-6) are plotted in Fig 2. It can be observed that the entire CC hydrogels (CCH-2, CCH-4 and CCH-6) exhibit a higher degree of swelling DS than the KCPH formed from untreated native cellulose. The degree of swelling increased with an increasing of nitrogen concentration in the CC hydrogels. This result is theorized to be due to the higher nitrogen concentration which disrupts the inter-molecular hydrogen bonding of the cellulose chain by introducing amide groups making the CC decrease in stiffness. Therefore, with the more flexible cellulose structure, water can penetrates into the cellulose hydrogel structure more easily and approaches equilibrium after a certain period of time. The SEM images (will be discussed later) show higher porosity for CC hydrogel with higher nitrogen content. This observation was reported by Ganji et al. [29] who explained that the swelling mechanism was primarily because of the diffusion of water that filled the pores in cellulose hydrogel.

### Transparency of cellulose hydrogels

**Fig 3:**
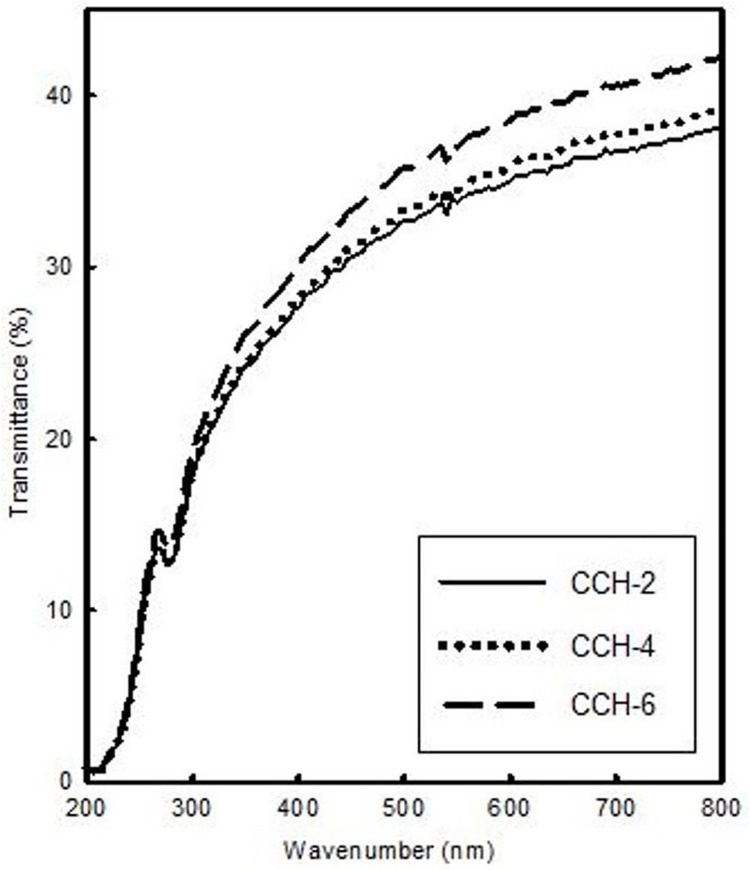
Transparency of regenerated hydrogel.

Optical transmittance is an auxiliary method to evaluate cellulose hydrogel quality. The light transmittance (%) through the regenerated cellulose hydrogels (CCH-2, CCH-4 and CCH-6) were characterized by UV–vis spectrophotometer with a wavelength range between 200 and 800 nm (Fig 3). From Fig 3, the transmittance (%) of CC hydrogels is as: CCH-6 > CCH-4 > CCH-2. It shows that as the nitrogen concentration in CC increases, the transmittance of its regenerated hydrogel also slightly increases. This could be ascribed the more open/porous structure of the hydrogels allowing transmittance of light.

### FT-IR analysis of cellulose carbamate and aerogels

**Fig 4:**
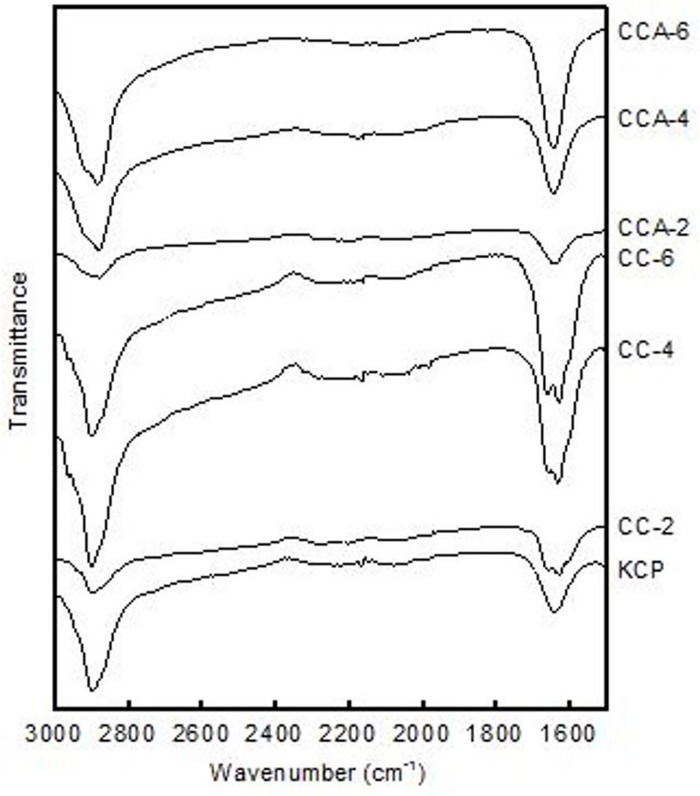
FT-IR spectra of CC and cellulose aerogels.

Fig 4 depicts the FT-IR spectra of native cellulose KCP, CC-2, CC-4, CC-6 and regenerated cellulose aerogel samples (CCA-2, CCA-4 and CCA-6). Each spectra show a peak at 2904 cm^-1^, assigned to N-H stretching in urea. Two peaks at 1665 cm^-1^ and 1628 cm^-1^ in CC are attributed to the stretching vibration of the carbonyl (C = O) in the amide group after cellulose carbamation [1, 13]. A peak at 1646 cm^-1^ referring to the absorption of water by cellulose [30] is observed in the KCP and regenerated cellulose aerogels (CCA-2, CCA-4 and CCA-6). The difference in the spectra of regenerated cellulose aerogels differs from its original CC due to the carbamate functional groups being partly cleaved off the CC backbone during the cellulose dissolution and regeneration processes [5].

### XRD patterns of cellulose aerogels

**Fig 5:**
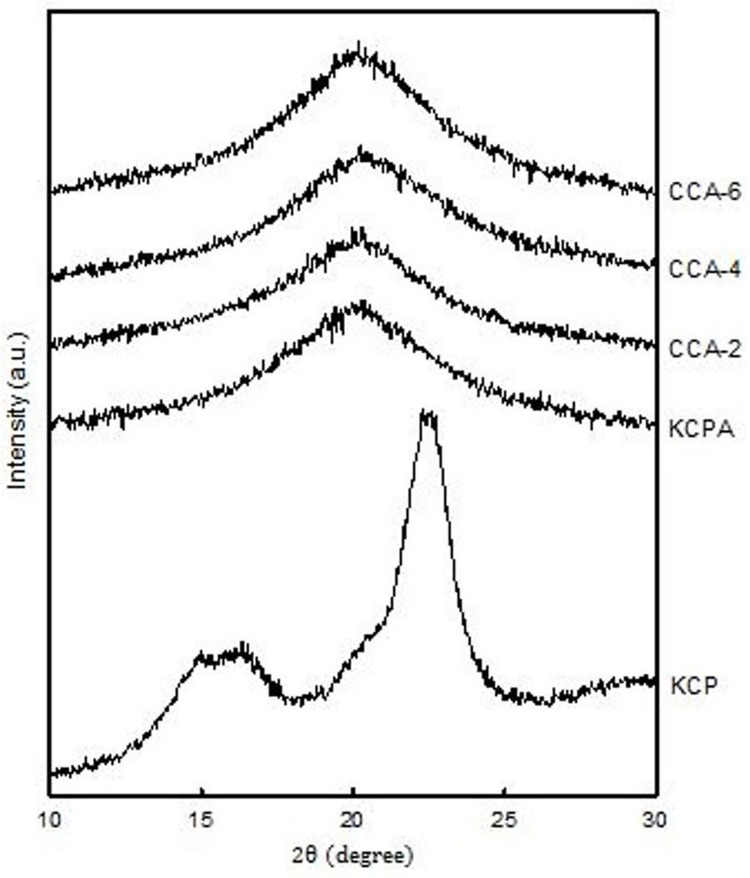
XRD patterns of regenerated aerogel.

Fig 5 illustrates the XRD diffraction patterns of native cellulose KCP and regenerated cellulose aerogels (KCPA, CCA-2, CCA-4 and CCA-6). The diffraction peaks of native cellulose KCP are located at 2θ = 14.9°, 16.2° and 22.5° and are referred to as the cellulose crystal planes of (1 ī 0), (1 1 0) and (2 0 0), respectively, as reported previously by [31]. Native cellulose tends to aggregate into highly organize crystalline structures due to its various types of bonding such as van der Waals, hydrophobic, and inter- and intramolecular hydrogen bonding [31]. The broad peak of all aerogel samples at 20.2° shows evidence of the formation of cellulose II in all the regenerated cellulose aerogels, as reported previously [32]. In addition, the broad peak illustrates the destruction of the cellulose crystalline structure during the formation of cellulose hydrogel which involves the breaking of hydrogen bonds. This process encourages the formation of amorphous area in cellulose hydrogel structure [33,34].

Upon cellulose dissolution and regeneration, the cellulose I peak in KCP shifts to reflect more of a cellulose II structure for all cellulose aerogel samples (KCPA, CCA-2, CCA-4 and CCA-6) as shown in Fig 5. The CrI (crystallinity index) of the regenerated cellulose aerogels cannot be analysed accurately due to the chemical cross-linking of cellulose with ECH upon the formation of cellulose hydrogel. This has resulted in the destruction of the initial crystalline structure of cellulose [35].

### Morphology of cellulose aerogels

**Fig 6:**
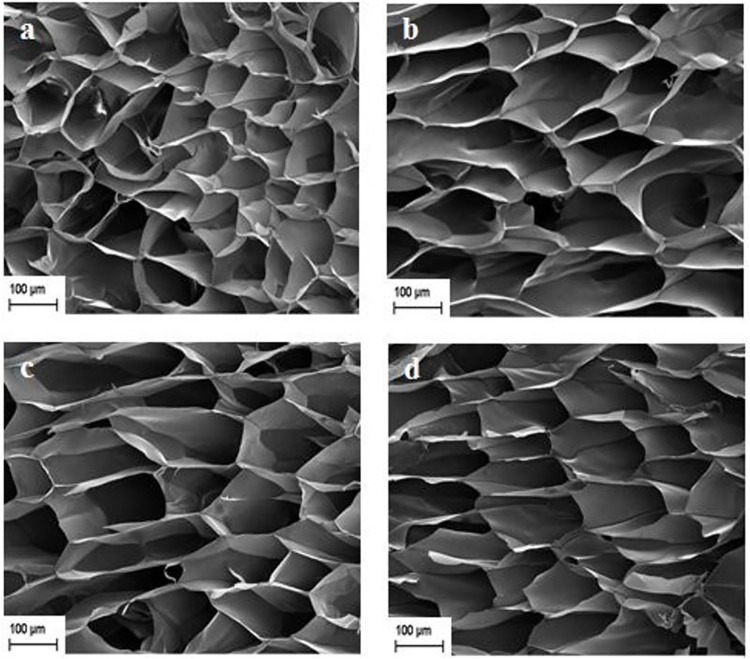
FESEM photographs of regenerated aerogel (a) KCPA (b) CCA-2 (c) CCA-4 and (d) CCA-6.

Fig 6 shows micrographs of (a) KCPA (b) CCA-2 (c) CCA-4 and (d) CCA-6. From the observation, all regenerated cellulose aerogels have macroporous structure. This can be explained as: during the freeze drying process, the trapped water in the cellulose hydrogel turns into ice crystals with phase separation followed by subsequent sublimation which causes the formation of voids in the cellulose aerogels. The pore size of the KCPA, CCA-2, CCA-4 and CCA-6 was 148 ± 57, 154 ± 65, 161 ± 72 and 168 ± 64 μm, respectively. It appears that the pore sizes of the aerogel samples are not significantly affected by the urea content. The introduction of the carbonyl and amine groups in the cellulose did not obviously effect on the porosity of its regenerated cellulose hydrogel although the additional functional groups might disturb the inter-molecular hydrogen bonding and creates a more flexible cellulose structure. Nonetheless, the slight increment of pore size allows more water to be retained in the cellulose hydrogels, as observed in the swelling results (Fig 2). This swelling then leads to an increase in the pore size of cellulose aerogels upon freeze-drying.

## Conclusions

Cellulose carbamate (CC) was successfully synthesized under hydrothermal condition without any catalyst and organic solvent. The formation of CC with the aid of hydrothermal has been verified through the FT-IR characterization and nitrogen concentration analysis. The nitrogen concentration obtained in CC increased with respect to the urea concentration used in the preparation of CC. The yield of CC endowed with high nitrogen concentration and possessed desirable solubility in urea alkaline solution. The XRD indicated the formation of cellulose II in cellulose aerogel prepared from CC solution. The nitrogen concentration obtained in CC and its solubility properties were discovered to have the corresponding effect on morphology, transparency and swelling properties of the regenerated CC materials (hydrogel and aerogel) in this study. As the supplying urea increased in the carbamation process, the degree of swelling, transparency and pore size of the cellulose hydrogel increased. The carbamate process is still under development and might essential in the future of regenerated cellulosic. Therefore, this carbamation process is expected useful in producing more environmental friendly regenerated cellulose products especially in the field of water absorbent.
